# Parent-reported quality of life in children with cochlear implants differs across countries

**DOI:** 10.3389/fpsyg.2022.966401

**Published:** 2022-10-06

**Authors:** Andrea D. Warner-Czyz, Jackie A. Nelson, Roshini Kumar, Sarah Crow

**Affiliations:** ^1^Children and Infant Listening Laboratory, Department of Speech, Language and Hearing, The University of Texas at Dallas, Richardson, TX, United States; ^2^Family Research Laboratory, Department of Psychology, The University of Texas at Dallas, Richardson, TX, United States; ^3^Center for Pediatric Psychiatry, Children’s Health, Dallas, TX, United States

**Keywords:** quality of life, cochlear implant, children, deaf and hard of hearing, international

## Abstract

Pediatric cochlear implantation affects communication skills and quality of life, specifically how children interact with others and feel about themselves. Numerous studies worldwide examine well-being among pediatric cochlear implant users, but none to date compare condition-specific quality of life across countries. This retrospective study compares parent-reported cochlear implant-specific quality of life summary data across 14 published studies spanning 11 countries and 9 languages. Sample size ranged from 7 to 370 participants, and children across studies varied in mean chronologic age (3.1–12.2 years), implantation age (1.5–4.6 years), and cochlear implant experience (1.3–8.2 years). Parents completed the Children with Cochlear Implants: Parental Perspectives (*CCIPP*) questionnaire, an instrument assessing parent-reported cochlear implant-specific quality of life, in their home language. Analysis of variance tests were run for each *CCIPP* subscale across studies using summary data to determine significant differences between published manuscripts. Across countries, parents of children with cochlear implants appraise communication, social relations, and self-reliance most positively, and the effects of implantation and supporting the child least positively. Cross-country analyses revealed a significant effect of study (country) on quality of life ratings in each domain, with the largest differences in the communication domain. Limited access to implant-related accommodations, cultural awareness of hearing loss, and varying parent expectations may explain country differences in parental ratings of quality of life. Culturally sensitive psychoeducation for the entire family may foster improved life satisfaction for pediatric cochlear implant users and their families.

## Introduction

Cochlear implantation in children who are deaf/hard of hearing (DHH) affects not only communication skills such as speech recognition, speech production, and language use, but also how these children interact with others and how they feel about themselves—also known as psychosocial well-being, a component of quality of life (QoL; e.g., Kouwenberg et al., 2012; Huber et al., 2015; Theunissen et al., 2015; Warner-Czyz et al., 2018). Because pediatric cochlear implant (CI) users may not have the language skills to convey their QoL, parents can serve as a reliable proxy to assess their child’s well-being across multiple domains. However, several factors may influence a parent’s appraisal of their child’s QoL, including demographic characteristics of the child (e.g., chronologic age, presence of additional disabilities), cochlear implant-related factors (e.g., age at implantation, duration of device use), the aspect of well-being measured (e.g., communication versus self-esteem), family dynamics, and societal and cultural norms relative to hearing levels and cochlear implantation. The present study adopts a global perspective to conduct a comparison of parent-reported QoL in pediatric CI recipients across countries.

### Assessing QoL in children with CIs

QoL assessment tools can either be generic or condition-specific. Generic QoL instruments assess physical, psychological, and social well-being independent of a medical condition. Generic measures of QoL typically yield comparable ratings among children with CIs and typical hearing peers on overall QoL and physical well-being, but differences have been found in the domains of psychological and social well-being (Huber, 2005; Loy et al., 2010; Theunissen et al., 2014; Warner-Czyz et al., 2018). Generic QoL instruments allow comparison of QoL ratings across various diseases, interventions, or conditions, including comparison with neurotypical, healthy populations (e.g., children using CIs vs. children with typical hearing). The major drawback of a generic measure of QoL is its insensitivity to smaller changes specific to a treatment, condition, or population (Lin and Niparko, 2006).

Alternatively, condition-specific instruments have greater sensitivity to positive and negative aspects of a particular treatment or condition (Bjornson and McLaughlin, 2001). Validated condition-specific questionnaires are common for chronic conditions such as cancer and diabetes but sparse for other conditions such as cochlear implantation. The last 25 years has seen the development of several CI-specific QoL measures for children (presented here in chronologic order): *Survey of Parents of Pediatric Cochlear Implantees* (Gallaudet Research Institute, 1999), *Cochlear Implant Expectations Questionnaire* (Zaidman-Zait and Most, 2005); *Children with Cochlear Implants: Parental Perspectives* (*CCIPP*; Archbold et al., 2008), *Parent Expectations Questionnaire for Cochlear Implants* (Nemours Children’s Clinic, 2010), *Parental Attitudes of Various Aspects of Cochlear Implantation* (Soleimanifar et al., 2015), *Brief Assessment of Parental Perception* (Samuel et al., 2016), and *Quality of Life—Cochlear Implant* (Cejas et al., 2021). All these measures use parent proxy reports, in which parents appraise their child’s QoL; only one instrument (*Quality of Life—Cochlear Implant*) also has a self-report option for pediatric CI users to rate their own well-being relative to cochlear implantation. Fewer than five studies report on parent proxy QoL outcomes using each of the following measures: *Survey of Parents of Pediatric Cochlear Implantees* (Christiansen and Leigh, 2004; Hyde et al., 2010); the *Cochlear Implant Expectations Questionnaire* (Zaidman-Zait and Most, 2005; Hyde et al., 2010; Punch and Hyde, 2010), *Parent Expectations Questionnaire for Cochlear Implants* (Kumar et al., 2016; Alam et al., 2019; Alkhatani, 2021; Halawani et al., 2021), and the *Quality of Life—Cochlear Implant* (Hoffman et al., 2019; Cejas et al., 2021). The *CCIPP* reflects the most frequently used CI-specific QoL instrument worldwide (e.g., Incesulu et al., 2003; Nicholas and Geers, 2003; O’Neill et al., 2004; Nunes et al., 2005; Archbold et al., 2006, 2008; Damen et al., 2007; Huttunen et al., 2009; Fortunato-Tavares et al., 2012; Sparreboom et al., 2012; Stefanini et al., 2014; de Almeida et al., 2015; Kumar et al., 2015; Yorgun et al., 2015; Asfour et al., 2018; Byckova et al., 2018; Zhao et al., 2018; Hassuji, 2019; Molla et al., 2019; Tokat et al., 2019; Brewis et al., 2020; Peker et al., 2020; Shahmahmood et al., 2020; Silva et al., 2020, 2021; Anne et al., 2021; Yang et al., 2022; Zhang et al., 2022; Zhumabayev et al., 2022).

Parent ratings of their child’s QoL can provide a well-rounded overview of the child’s well-being. Still, parent and child assessment of QoL do not always align. The accuracy of parent proxy assessment of a child’s well-being often depends on the dynamics of the parent–child relationship such that closer attachments coincide with closer parental attunement to their child’s needs and wellness (Ainsworth, 1967; Ma and Huebner, 2008). In general, parents tend to provide reliable responses for observable behaviors such as physical function and family relational domains (e.g., inter-family dynamics and sibling relationships; Eiser, 1997; Eiser and Morse, 2001; Eiser and Jenney, 2007). Parent ratings may be less reliable for cognitive and emotional attributes, including judgments, peer relationships, and future worries (Eiser and Morse, 2001; Huber, 2005; Eiser and Jenney, 2007; Warner-Czyz et al., 2009; Loy et al., 2010). However, some children cannot self-report their QoL due to illness severity, fatigue, or underdeveloped communication skills that preclude provision of an accurate description of their feelings or QoL (Hays et al., 2006). As the closest proxy to a child, parents can effectively answer various questions about QoL as it relates to their child’s well-being (Varni et al., 2007; Dodson et al., 2008).

### Parent ratings of condition-specific QoL in children with CIs

Regardless of the instrument used, parent proxy ratings of CI-specific QoL in children converge on the positive impact of cochlear implantation on their child’s general well-being (Warner-Czyz et al., 2009; Loy et al., 2010; Yorgun et al., 2015), as well as on specific domains such as communication, social relations, and self-reliance. For example, multiple studies report speech and language development as the largest and most consistent change in their child after implantation (e.g., Incesulu et al., 2003; Sach and Whynes, 2005; Archbold et al., 2008; Huttunen et al., 2009; Zaidman-Zait et al., 2015; Byckova et al., 2018; Molla et al., 2019; Brewis et al., 2020), though parents still may express concerns about their child’s speech and language development relative to peers with typical hearing (Archbold et al., 2008). The effect of cochlear implantation on a child’s social interactions (Incesulu et al., 2003; Sach and Whynes, 2005; Archbold et al., 2008; Huttunen et al., 2009) and independence (Incesulu et al., 2003; Sach and Whynes, 2005; Archbold et al., 2008; Huttunen et al., 2009) consistently garner positive ratings from parents. Parents in some studies link the two domains, positing that cochlear implantation positively affected their children’s well-being and self-reliance, which contributes to an inclination to become more involved in social groups and clubs (Sach and Whynes, 2005; Archbold et al., 2008).

In general, QoL domains generating greater concern from parents of CI users across studies include education and academic achievement (Hyde et al., 2010; Zhao et al., 2018; Bhamjee et al., 2019) and family support (Sach and Whynes, 2005; Stefanini et al., 2014; Zaidman-Zait et al., 2015; Dev et al., 2018; Scarinci et al., 2018), though not all studies agree with this assessment (Damen et al., 2007; Byckova et al., 2018).

Certain demographic characteristics may affect parental ratings of QoL in their children with CIs. Younger chronologic age, younger age at intervention, and younger age at implantation generally coincide with more positive QoL (Huber, 2005; Schorr et al., 2009; Warner-Czyz et al., 2009; Loy et al., 2010; Alkhamra, 2015; Alkhatani, 2021), although Silva et al. (2020) report more positive QoL with older chronologic age. Longer duration of CI experience also correlates with more positive QoL compared to shorter duration of CI experience (Huber, 2005; Clark et al., 2012; Yorgun et al., 2015; Zhao et al., 2018; Brewis et al., 2020). Use of oral communication rather than total communication or sign language corresponds to higher ratings of QoL in children using CIs (Fortunato-Tavares et al., 2012). Also, use of bilateral CIs or bimodal input (i.e., CI with a hearing aid on the contralateral side) enhances communication outcomes in children relative to unilateral implantation (Ching et al., 2001; Galvin and Mok, 2015). Finally, parents across studies indicate the presence of additional comorbidities can affect their overall appraisal of QoL, as well as specific domains such as supporting the child and independence (Bhamjee et al., 2019; Anne et al., 2021). However, other studies report no significant correlations between child demographic characteristics and parent-reported QoL (de Almeida et al., 2015; Kumar et al., 2015; Khan and Rajguru, 2021).

Synthesis of these studies provides a general understanding of the impact of cochlear implantation on pediatric QoL. However, these studies use a variety of QoL instruments with different items, domains, and purposes, which prohibits an accurate comparison of outcomes across samples using the same measure.

### Usefulness of comparison of QoL in children with CIs across countries

Similarities and differences in parental perspectives of child QoL across studies may reflect not only child-related factors, but also variables associated with lifestyle, parenting, or access to implant-specific resources across countries. For example, countries differ in areas such as accessibility of early hearing detection and intervention programs (e.g., age at identification as deaf/hard of hearing, age at cochlear implantation); cost of cochlear implant-related expenses (e.g., governmental coverage of devices, therapeutic intervention); and availability of accommodations (e.g., communication among members of the cochlear implant team, the school, and the family to identify and attain appropriate resources). Furthermore, differences may stem from cultural factors such as presence of a multilingual community (i.e., different rates of development in each language); the importance of self (i.e., individualism) versus the importance of the group (i.e., collectivism); and overall life satisfaction within that country (i.e., the United Nations consistently ranks Northern European countries as the world’s happiest countries; Hofstede, 2003). Country-based comparisons offer insight into dynamics that can influence a child’s functioning and satisfaction levels. Differences in QoL across countries may reflect cultural perspectives relative to acceptance of CI and hearing access, parenting style, or socio-economic status.

### Purpose of this study

The literature supports a considerable amount of studies examining QoL in children using CIs, but these studies include vast variability in both the well-being instrument and the sample characteristics reported. No published studies to date compare condition-specific QoL of pediatric CI users across countries. The present study addresses this gap in the growing literature conducted around the world by comparing parent reports of cochlear implant-specific QoL in pediatric CI users using the same instrument across multiple countries and languages. This study uses a retrospective study design analyzing summary statistic data from previously published reports to ask the following research question: Does condition-specific QoL of pediatric cochlear implant users significantly differ across studies conducted around the world on the following established dimensions: communication, general functioning, self-reliance, well-being, social relations, education, effects of implantation, and supporting the child.

## Materials and methods

### Study selection

The authors searched multiple scientific databases (e.g., CINAHL, EBSCO Databases POWERSEARCH, Medline, PubMed, Web of Science) using the following search terms: children or adolescents or youth or child or teenager; cochlear implant or cochlear implants or cochlear implantation; quality of life or well being or well-being or health-related quality of life or qol or hrqol.

Ninety-five studies conducted around the world were assessed for eligibility to test differences in parent-reported condition-specific QoL in pediatric cochlear implant recipients. Studies needed to meet the following criteria: (a) Use of the Archbold et al. (2008) *CCIPP* questionnaire; (b) Inclusion of descriptive statistics (i.e., sample size, mean, and standard deviation) for domain-specific scores; and (c) publication in English. The first author contacted the lead or corresponding author on 12 published papers that used the *CCIPP* questionnaire but did not report domain-specific outcomes on a five-point scale for each of the eight domains to collect descriptive statistics necessary for data analysis. Four authors provided supplementary summary data to include their manuscript in the analyses (Huttunen et al., 2009; Fortunato-Tavares et al., 2012; de Almeida et al., 2015; Shahmahmood et al., 2020). In addition, the first author calculated descriptive statistics for QoL domains based on detailed tables provided in the manuscripts for five additional manuscripts (Damen et al., 2007; Archbold et al., 2008; Stefanini et al., 2014; Byckova et al., 2018; Molla et al., 2019).The authors excluded 81 studies due to use of a different QoL measure (*n* = 68, including 23 papers using *ad hoc* questionnaires, 8 conducting semi-structured interviews, 17 administering hearing loss-specific measures, and 20 utilizing alternative CI-specific measures); or use of a different version of Archbold et al.’s questionnaire published before 2008 (*n* = 3), use of the *CCIPP* questionnaire but reporting on different domains (i.e., decision-making and the process of implantation, *n* = 1) or not reporting specific domains scores (*n* = 7), administering to a different population (i.e., pediatric auditory brainstem implant recipients, *n* = 1), or publishing in a different language (i.e., Chinese, *n* = 1).

Fourteen published studies representing 9 languages and 11 countries met the inclusion criteria (Table 1). One study contributed two data points for groups differing in age at implantation (de Almeida et al., 2015) and a different study did not report scores for all eight domains (Byckova et al., 2018). Table 1 details the country and language of origin, sample size, and participant demographic characteristics.

**Table 1:** Summary of articles included in the analyses.

Author	Country/language	*n*	Mean chronologic age (years)	Mean age at CI (years)	Mean CI experience (years)	Communication approach
Anne et al. (2021)	United States/English	35	8.5 (*SD* = 3.6)	3.5 (*SD* = 3.5)	4.9 (*SD* = 2.3)	
Archbold et al. (2008)	United Kingdom/English	101		4.7; Range: 1.3–12.4	3	59% oral, 39% sign-supported, 2% sign
Brewis et al. (2020)	South Africa/English	54	12.2 (*SD* = 3.6); Range: 6.6–18.3	3.9 (*SD* = 2.4); Range: 0.6–11.6	8.2 (*SD* = 4.1); Range: 0.7–15.8	80% oral, 20% total communication
Byckova et al., 2018	Lithuania/Lithuanian	28	6.1 (*SD* = 3.3); Range: 3.5–18.7	2.4 (*SD* = 2.3); Range: 1.1–11.1	3.7 (*SD* = 1.3); Range: 2.3–7.6	100% oral
Damen et al. (2007)	Netherlands/Dutch	130	8.9; Range: 1.9–18.2	4.6; Range: 0.7–14.3		57% oral, 34% sign-supported, 9% sign
de Almeida et al. (2015), CI < 24 months	Brazil/Brazilian Portuguese	7	7.5; Range: 1.5–12.2	4.5; Range: 0.9–8.2	1.3 (*SD* = 0.6); Range ≥ 0.5	100% oral
de Almeida et al. (2015), CI > 24 months		8			4.5 (*SD* = 1.6); Range ≥ 0.5	100% oral
Fortunato-Tavares et al. (2012)	Brazil/Brazilian Portuguese	10	6.2 (*SD* = 2.5)	4.5 (SD = 2.2)	1.5 (*SD* = 0.8)	
Huttunen et al. (2009)	Finland/Finnish	36	5.0 (*SD* = 2.0); Range: 3.0–15.0	3.4 (*SD* = 2.3); Range: 1.5–12.3	2–3 years	67% oral, 22% total communication, 11% sign
Kumar et al. (2015)	United States/English	33	9.9 (*SD* = 3.3); Range 4.0–18.0	2.5 (*SD* = 1.9); Range: <1.0–8.0	7.5 (*SD* = 2.8); Range: 1–12	100% oral
Molla et al. (2019)	Bangladesh/Bangla	25	7.2 (*SD* = 1.5); Range: 5.0–10.0	3.6 (*SD* = 1.1); Range: 1.0–6.0	3.6 (*SD* = 1.3); Range: 2.0–6.0	
Shahmahmood et al. (2020)	Iran/Persian	370	3.0-7.0	1.5 (0.3)	4.4 (3.0); Range: ≥1.5	
Stefanini et al. (2014)	Brazil/Brazilian Portuguese	50	4.3	2.2	~2	
Zhao et al. (2018)	China/Mandarin	51	3.1 (*SD* = 1.2); Range: 1.7–6.8	2.1 (*SD* = 1.2); Range: 0.7–4.9	≥1	
Zhumabayev et al. (2022)	Kazakhstan/Kazakh or Russian	53	7.3 (*SD* = 3.3); Range: 2.8–15.0	3.2 (*SD* = 1.6); Range: 1.0–7.0	4.2 (*SD* = 2.5); Range: 1.0–12.0	

de Almeida et al. (2015) did not provide group-specific demographic statistics.

### Materials

All published manuscripts included in the analyses included completion of the *CCIPP* questionnaire (Archbold et al., 2008), a validated parental proxy assessment of CI-specific QoL. The topics and items of this questionnaire emerged from parental experiences as described in their own words through either an open-ended questionnaire or an interview (Archbold et al., 2002). The resulting questionnaire includes 74 items covering two overarching domains of the process of implantation: decision-making (26 items) and outcomes (48 items). Specifically, the outcomes of implantation domain has eight subscales, including communication (6 items), general functioning (6 items), well-being (5 items), self-reliance (4 items), social relations (7 items), education (7 items), effects of implantation (7 items), and supporting the child (6 items; Archbold et al., 2008).

Parents can complete the questionnaire as either a semi-structured interview or as a self-administered survey. Items from each domain are dispersed throughout the questionnaire to not group statements according to a theme. Parents rate their response to each statement on a five-point Likert scale that ranges from strongly agree to strongly disagree. The questionnaire’s items are balanced for negativity and positivity, with 46 statements phrased in a positive format (e.g., “Now s/he is talkative and engages others in conversation”) and 28 statements in a negative format (e.g., “S/he is unable to cope with mainstream schooling”; Archbold et al., 2008). Scoring of negative statements gets reversed so that a higher value indicates a more positive response. Several studies have established the *CCIPP* as a valid and reliable measure to assess parents’ perceptions of how pediatric cochlear implants affect children’s lives (O’Neill et al., 2004; Nunes et al., 2005).

### Statistical analyses

Even when raw data are unavailable, analyses of variance (ANOVA) can be used to compare three or more groups using the summary statistics of sample mean, sample standard deviation, and number of participants (Larson, 1992). Descriptive statistics were collated for all variables of interest. ANOVA tests were run using summary data from the included manuscripts. A separate ANOVA was conducted for each subscale of the *CCIPP*. A *p*-value < 0.05 suggests a significant difference exists between the various samples. This omnibus test is followed by Tukey HSD *post-hoc* tests to determine which specific pairs of samples differ significantly from each other. ANOVA with summary statistics can be run in many statistical packages and with the following online calculator that was used in the current study for the omnibus and *post-hoc* tests.^1^

## Results

### Collation of descriptive statistics across published studies

Means and standard deviations were collated across the published studies (displayed in Table 2). Three samples from two studies (Fortunato-Tavares et al., 2012; de Almeida et al. 2015) reported means and standard deviations on a-100 to 100 scale analyzed by the Parent Views and Experiences Questionnaire Data Entry (version 1.02: ISVR Software, Copyright 2003) prepared by the Ear Foundation team. We rescaled these values to a 0 to 5 scale.

**Table 2:** Means and standard deviations by article and subscale.

Author	Communication	General functioning	Self-reliance	Well-being	Social relations	Education	Effects of implantation	Supporting the child
Anne et al. (2021)	4.22 (0.57)	3.88 (0.43)	3.68 (0.46)	3.97 (0.47)	3.97 (0.47)	4.17 (0.53)	3.55 (0.59)	3.27 (0.47)
Archbold et al. (2008)	3.94 (0.39)	3.62 (0.93)	3.34 (1.01)	3.65 (0.69)	3.81 (0.70)	3.68 (0.50)	3.48 (0.50)	3.03 (0.90)
Brewis et al. (2020)	4.15 (0.62)	4.05 (0.51)	3.88 (0.63)	3.81 (0.60)	3.87 (0.52)	3.70 (0.64)	3.49 (0.62)	3.46 (0.47)
Byckova et al., 2018	3.90 (0.77)	−	3.30 (0.27)	−	4.05 (0.41)	−	−	3.89 (0.49)
Damen et al. (2007)	3.94 (0.21)	3.56 (0.84)	3.44 (0.69)	3.58 (0.58)	3.79 (0.45)	3.73 (0.39)	3.69 (0.60)	3.13 (0.88)
de Almeida et al. (2015), CI < 24 months	3.69 (0.27)	3.43 (0.30)	3.93 (0.41)	2.96 (0.23)	3.84 (0.20)	3.40 (0.25)	2.77 (0.16)	2.28 (0.30)
de Almeida et al. (2015), CI > 24 months	2.97 (0.35)	3.59 (0.38)	4.01 (0.22)	3.19 (0.16)	3.98 (0.34)	2.91 (0.19)	2.64 (0.25)	2.23 (0.40)
Fortunato-Tavares et al. (2012)	2.81 (0.47)	3.06 (0.38)	3.49 (0.31)	3.35 (0.25)	3.41 (0.25)	2.81 (0.27)	2.75 (0.23)	2.19 (0.33)
Huttunen et al. (2009)	4.30 (0.77)	4.27 (0.46)	4.27 (0.63)	3.91 (0.42)	4.33 (0.49)	3.82 (0.58)	3.68 (0.68)	3.81 (0.60)
Kumar et al. (2015)	3.90 (0.62)	3.90 (0.50)	3.70 (0.80)	3.70 (0.62)	3.90 (0.40)	3.30 (0.50)	3.11 (0.70)	3.70 (0.60)
Molla et al. (2019)	3.18 (0.65)	3.05 (1.15)	2.92 (1.29)	2.96 (1.37)	3.63 (0.92)	3.29 (0.84)	2.81 (1.11)	2.49 (0.95)
Shahmahmood et al. (2020)	3.57 (0.64)	4.18 (0.53)	3.53 (0.55)	4.09 (0.52)	3.62 (0.41)	3.75 (0.50)	3.81 (0.62)	3.20 (0.58)
Stefanini et al. (2014)	3.82 (0.52)	3.45 (0.95)	3.66 (0.65)	3.55 (0.88)	3.93 (0.27)	3.45 (1.01)	3.07 (1.02)	3.08 (0.89)
Zhao et al. (2018)	2.13 (0.58)	2.79 (0.44)	2.94 (0.37)	3.14 (0.34)	3.24 (0.42)	2.96 (0.34)	3.02 (0.28)	3.13 (0.31)
Zhumabayev et al. (2022)	3.58 (0.73)	3.57 (0.51)	3.80 (0.64)	3.89 (0.50)	3.85 (0.52)	3.48 (0.45)	3.15 (0.60)	3.87 (0.48)

Byckova et al. (2018) did not provide values for four domains.

Overall, 84% of mean parental ratings of QoL exceeded a value of 3, indicating neutral to positive appraisals of well-being across subscales. In general, the domains of Communication and Social Relations received the most positive ratings from parents, consistently ranking in the top two positive domains for QoL. Effects of Implantation and Supporting the Child yielded the least positive scores across studies, ranking as one of the two lowest scores in 10 and 11 studies, respectively.

### Comparisons of QoL

Supplementary Figures 1–8 show means plots for each subscale. For the Communication subscale, the omnibus ANOVA test was significant, *F*(14,976) = 43.26, *p* < 0.001. Table 3 shows pairwise mean differences between samples. Fortunato-Tavares et al. (2012), de Almeida et al. (2015; <24 months), Molla et al. (2019), and Zhao et al. (2018) tended to report lower mean ratings for the Communication subscale compared to other samples.

**Table 3:** Communication subscale Tukey HSD *post-hoc* mean differences.

	Anne et al. (2021)	Archbold et al. (2008)	Brewis et al. (2020)	Byckova et al., 2018	Damen et al. (2007)	de Almeida et al. (2015) < 24 months	de Almeida et al. (2015) > 24 months	Fortunato-Tavares et al. (2012)	Huttunen et al. (2009)	Kumar et al. (2015)	Molla et al. (2019)	Shahmahmood et al. (2020)	Stefanini et al. (2014)	Zhao et al. (2018)
Archbold et al. (2008)	−0.28													
Brewis et al. (2020)	−0.07	0.21												
Byckova et al., 2018	−0.32	−0.04	−0.25											
Damen et al. (2007)	−0.28	0.00	−0.21	0.04										
de Almeida et al. (2015) < 24 months	−0.53	−0.25	−0.46	−0.21	−0.25									
de Almeida et al. (2015) >24 months	−1.25^***^	−0.97^***^	−1.18^***^	−0.93^**^	−0.97^***^	−0.72								
Fortunato-Tavares et al. (2012)	−1.41^***^	−1.13^***^	−1.34^***^	−1.09^***^	−1.13^***^	−0.88	−0.16							
Huttunen et al. (2009)	0.08	0.36	0.15	0.40	0.36	0.61	1.33^***^	1.49^***^						
Kumar et al. (2015)	−0.32	−0.04	−0.25	0.00	−0.04	0.21	0.93^**^	1.09^***^	−0.40					
Molla et al. (2019)	−1.04^***^	−0.76^***^	−0.97^***^	−0.72^***^	−0.76^***^	−0.51	0.21	0.37	−1.12^***^	−0.72^***^				
Shahmahmood et al. (2020)	−0.65^***^	−0.37^***^	−0.58^***^	−0.33	−0.37^***^	−0.12	0.60	0.76^**^	−0.73^***^	−0.33	0.39			
Stefanini et al. (2014)	−0.40	−0.12	−0.33	−0.08	−0.12	0.13	0.85^**^	1.01***	−0.48^*^	−0.08	0.64^***^	0.25		
Zhao et al., 2018	−2.09^***^	−1.81^***^	−2.02^***^	−1.77^***^	−1.81^***^	−1.56^***^	−0.84^*^	−0.68^*^	−2.17^***^	−1.77^***^	−1.05^***^	−1.44^***^	−1.69^***^	
Zhumabayev et al. (2022)	−0.64^***^	−0.36^*^	−0.57^***^	−0.32	−0.36^*^	−0.11	0.61	0.77^**^	−0.72^***^	−0.32	0.40	0.01	−0.24	1.45^***^

Mean differences are reported as row minus column.

* *p* < 0.05;

** *p* < 0.01;

*** *p* < 0.001.

For the General Functioning subscale, the omnibus ANOVA test was significant, *F*(13,949) = 27.48, *p* < 0.001. Pairwise mean differences between samples are shown in Table 4. Molla et al. (2019) and Zhao et al. (2018) tended toward lower scores compared to some of the other samples, and Huttunen et al. (2009) and Shahmahmood et al. (2020) tended toward higher scores than the other samples.

**Table 4:** General functioning subscale Tukey HSD *post-hoc* mean differences.

	Anne et al. (2021)	Archbold et al. (2008)	Brewis et al. (2020)	Damen et al. (2007)	de Almeida et al. (2015) < 24 months	de Almeida et al. (2015) > 24 months	Fortunato-Tavares et al. (2012)	Huttunen et al. (2009)	Kumar et al. (2015)	Molla et al. (2019)	Shahmahmood et al. (2020)	Stefanini et al. (2014)	Zhao et al. (2018)
Archbold et al. (2008)	−0.26												
Brewis et al. (2020)	0.17	0.43^**^											
Damen et al. (2007)	−0.32	−0.06	−0.49^***^										
de Almeida et al. (2015) < 24 months	−0.45	−0.19	−0.62	−0.13									
de Almeida et al. (2015) > 24 months	−0.29	−0.03	−0.46	0.03	0.16								
Fortunato-Tavares et al. (2012)	−0.82^*^	−0.56	−0.99^**^	−0.50	−0.37	−0.53							
Huttunen et al. (2009)	0.39	0.65^***^	0.22	0.71^***^	0.84	0.68	1.21^***^						
Kumar et al. (2015)	0.02	0.28	−0.15	0.34	0.47	0.31	0.84^*^	−0.37					
Molla et al., 2019	−0.83^***^	−0.57^**^	−1.00^***^	−0.51^*^	−0.38	−0.54	−0.01	−1.22^***^	−0.85^**^				
Shahmahmood et al. (2020)	0.30	0.56^***^	0.13	0.62^***^	0.75	0.59	1.12^***^	−0.09	0.28	1.13^***^			
Stefanini et al. (2014)	−0.43	−0.17	−0.60^***^	−0.11	0.02	−0.14	0.39	−0.82^***^	−0.45	0.40	−0.73^***^		
Zhao et al., 2018	−1.09^***^	−0.83^***^	−1.26^***^	−0.77^***^	−0.64	−0.80	−0.27	−1.48^***^	−1.11^***^	−0.26	−1.39^***^	−0.66^***^	
Zhumabayev et al. (2022)	−0.31	−0.05	−0.48^*^	0.01	0.14	−0.02	0.51	−0.70^***^	−0.33	0.52	−0.61^***^	0.12	0.78^***^

Mean differences are reported as row minus column.

* *p* < 0.05;

** *p* < 0.01;

*** *p* < 0.001.

For the Self-Reliance subscale, the omnibus ANOVA test was significant, *F*(14,976) = 11.17, *p* < 0.001. Table 5 displays pairwise mean differences between samples. Values reported in Molla et al. (2019) and Zhao et al. (2018) tended to be less positive than many of the other samples and Huttunen et al. (2009) tended to be more positive.

**Table 5:** Self-reliance subscale Tukey HSD *post-hoc* mean differences.

	Anne et al. (2021)	Archbold et al. (2008)	Brewis et al. (2020)	Byckova et al., 2018	Damen et al. (2007)	de Almeida et al. (2015) < 24 months	de Almeida et al. (2015) > 24 months	Fortunato-Tavares et al. (2012)	Huttunen et al. (2009)	Kumar et al. (2015)	Molla et al. (2019)	Shahmahmood et al. (2020)	Stefanini et al. (2014)	Zhao et al. (2018)
Archbold et al. (2008)	−0.34													
Brewis et al. (2020)	0.20	0.54^***^												
Byckova et al., 2018	−0.38	−0.04	−0.58^*^											
Damen et al. (2007)	−0.24	0.10	−0.44^**^	0.14										
de Almeida et al. (2015) < 24 months	0.25	0.59	0.05	0.63	0.49									
de Almeida et al. (2015) > 24 months	0.33	0.67	0.13	0.71	0.57	0.08								
Fortunato-Tavares et al. (2012)	−0.19	0.15	−0.39	0.19	0.05	−0.44	−0.52							
Huttunen et al. (2009)	0.59^*^	0.93^***^	0.39	0.97^***^	0.83^***^	0.34	0.26	0.78						
Kumar et al. (2015)	0.02	0.36	−0.18	0.40	0.26	−0.23	−0.31	0.21	−0.57^*^					
Molla et al. (2019)	−0.76^**^	−0.42	−0.96^***^	−0.38	−0.52^*^	−1.01^*^	−1.09^**^	−0.57	−1.35^***^	−0.78^**^				
Shahmahmood et al. (2020)	−0.15	0.19	−0.35^*^	0.23	0.09	−0.40	−0.48	0.04	−0.74^***^	−0.17	0.61^***^			
Stefanini et al. (2014)	−0.02	0.32	−0.22	0.36	0.22	−0.27	−0.35	0.17	−0.61^**^	−0.04	0.74^**^	0.13		
Zhao et al., 2018	−0.74^***^	−0.40^*^	−0.94^***^	−0.36	−0.50^***^	−0.99^*^	−1.07^**^	−0.55	−1.33^***^	−0.76^***^	0.02	−0.59^***^	−0.72^***^	
Zhumabayev et al. (2022)	0.12	0.46^**^	−0.08	0.50	0.36	−0.13	−0.21	0.31	−0.47	0.10	0.88^***^	0.27	0.14	0.86^***^

Mean differences are reported as row minus column.

* *p* < 0.05;

** *p* < 0.01;

*** *p* < 0.001.

For the Well-Being subscale, the omnibus ANOVA test was significant, *F*(13,949) = 20.43, *p* < 0.001. Pairwise mean differences between samples, shown in Table 6, indicate less positive ratings of well-being by Molla et al. (2019) and Zhao et al. (2018) and more positive ratings by Shahmahmood et al. (2020) compared to some of the other samples.

**Table 6:** Well-being subscale Tukey HSD *post-hoc* mean differences.

	Anne et al. (2021)	Archbold et al. (2008)	Brewis et al. (2020)	Damen et al. (2007)	de Almeida et al. (2015) < 24 months	de Almeida et al. (2015) > 24 months	Fortunato-Tavares et al. (2012)	Huttunen et al. (2009)	Kumar et al. (2015)	Molla et al. (2019)	Shahmahmood et al. (2020)	Stefanini et al. (2014)	Zhao et al. (2018)
Archbold et al. (2008)	−0.32												
Brewis et al. (2020)	−0.16	0.16											
Damen et al. (2007)	−0.39^*^	−0.07	−0.23										
de Almeida et al. (2015) < 24 months	−1.01^**^	−0.69	−0.85^*^	−0.62									
de Almeida et al. (2015) > 24 months	−0.78	−0.46	−0.62	−0.39	0.23								
Fortunato-Tavares et al. (2012)	−0.62	−0.30	−0.46	−0.23	0.39	0.16							
Huttunen et al. (2009)	−0.06	0.26	0.10	0.33	0.95**	0.72	0.56						
Kumar et al. (2015)	−0.27	0.05	−0.11	0.12	0.74	0.51	0.35	−0.21					
Molla et al. (2019)	−1.01^***^	−0.69^***^	−0.85^***^	−0.62^***^	0.00	−0.23	−0.39	−0.95^***^	−0.74^***^				
Shahmahmood et al. (2020)	0.12	0.44^***^	0.28	0.51^***^	1.13^***^	0.90^**^	0.74^**^	0.18	0.39^*^	1.13^***^			
Stefanini et al. (2014)	−0.42	−0.10	−0.26	−0.03	0.59	0.36	0.20	−0.36	−0.15	0.59^**^	−0.54^***^		
Zhao et al. (2018)	−0.83^***^	−0.51^***^	−0.67^***^	−0.44^***^	0.18	−0.05	−0.21	−0.77^***^	−0.56^**^	0.18	−0.95^***^	−0.41^*^	
Zhumabayev et al. (2022)	−0.08	0.24	0.08	0.31	0.93^**^	0.70	0.54	−0.02	0.19	0.93^***^	−0.20	0.34	0.75^***^

Mean differences are reported as row minus column.

* *p* < 0.05;

** *p* < 0.01;

*** *p* < 0.001.

For the Social Relations subscale, the omnibus ANOVA test was significant, *F*(14,976) = 13.01, *p* < 0.001. Pairwise mean differences between samples are shown in Table 7. Zhao et al. (2018) tended to appraise social relations less positively than the other samples and Huttunen et al. (2009) tended to rate this subscale more positively than the other samples.

**Table 7:** Social relations subscale Tukey HSD *post-hoc* mean differences.

	Anne et al. (2021)	Archbold et al. (2008)	Brewis et al. (2020)	Byckova et al., 2018	Damen et al. (2007)	de Almeida et al. (2015) < 24 months	de Almeida et al. (2015) > 24 months	Fortunato-Tavares et al. (2012)	Huttunen et al. (2009)	Kumar et al. (2015)	Molla et al. (2019)	Shahmahmood et al. (2020)	Stefanini et al. (2014)	Zhao et al. (2018)
Archbold et al. (2008)	−0.16													
Brewis et al. (2020)	−0.10	0.06												
Byckova et al., 2018	0.08	0.24	0.18											
Damen et al. (2007)	−0.18	−0.02	−0.08	−0.26										
de Almeida et al. (2015) < 24 months	−0.13	0.03	−0.03	−0.21	0.05									
de Almeida et al. (2015) >2 4 months	0.01	0.17	0.11	−0.07	0.19	0.14								
Fortunato-Tavares et al. (2012)	−0.56	−0.40	−0.46	−0.64^*^	−0.38	−0.43	−0.57							
Huttunen et al. (2009)	0.36	0.52^***^	0.46^***^	0.28	0.54^***^	0.49	0.35	0.92^***^						
Kumar et al. (2015)	−0.07	0.09	0.03	−0.15	0.11	0.06	−0.08	0.49	−0.43^*^					
Molla et al. (2019)	−0.34	−0.18	−0.24	−0.42	−0.16	−0.21	−0.35	0.22	−0.70^***^	−0.27				
Shahmahmood et al. (2020)	−0.35^**^	−0.19^*^	−0.25^*^	−0.43^***^	−0.17^*^	−0.22	−0.36	0.21	−0.71^***^	−0.28	−0.01			
Stefanini et al. (2014)	−0.04	0.12	0.06	−0.12	0.14	0.09	−0.05	0.52	−0.40^*^	0.03	0.30	0.31^**^		
Zhao et al. (2018)	−0.73^***^	−0.57^***^	−0.63^***^	−0.81^***^	−0.55^***^	−0.60	−0.74^**^	−0.17	−1.09^***^	−0.66^***^	−0.39	−0.38^***^	−0.69^***^	
Zhumabayev et al. (2022)	−0.12	0.04	−0.02	−0.20	0.06	0.01	−0.13	0.44	−0.48^***^	−0.05	0.22	0.23	−0.08	0.61^***^

Mean differences are reported as row minus column.

* *p* < 0.05;

** *p* < 0.01;

*** *p* < 0.001.

For the Education subscale, the omnibus ANOVA test was significant, *F*(13,949) = 16.62, *p* < 0.001. Table 8 shows pairwise mean differences between samples. Fortunato-Tavares et al. (2012), de Almeida et al. (2015; >24mo), and Zhao et al. (2018) tended to report less positive QoL than some of the other samples. Parents in Anne et al. (2021) tended to appraise Education more positively than the other samples. Damen et al. (2007), Huttunen et al. (2009), Archbold et al. (2008), Brewis et al. (2020), and Shahmahmood et al. (2020) tended to report more positive ratings of Education than Fortunato-Tavares et al. (2012), de Almeida et al. (2015; >24mo), Kumar et al. (2015), Zhao et al. (2018), and Molla et al. (2019).

**Table 8:** Education subscale Tukey HSD *post-hoc* mean differences.

	Anne et al. (2021)	Archbold et al. (2008)	Brewis et al. (2020)	Damen et al. (2007)	de Almeida et al. (2015) < 24 months	de Almeida et al. (2015) > 24 months	Fortunato-Tavares et al. (2012)	Huttunen et al. (2009)	Kumar et al. (2015)	Molla et al. (2019)	Shahmahmood et al. (2020)	Stefanini et al. (2014)	Zhao et al. (2018)
Archbold et al. (2008)	−0.49^***^												
Brewis et al. (2020)	−0.47^**^	0.02											
Damen et al. (2007)	−0.44^**^	0.05	0.03										
de Almeida et al. (2015) < 24 months	−0.77^*^	−0.28	−0.30	−0.33									
de Almeida et al. (2015) > 24 months	−1.26^***^	−0.77^**^	−0.79^**^	−0.82^**^	−0.49								
Fortunato-Tavares et al. (2012)	−1.36^***^	−0.87^***^	−0.89^***^	−0.92^***^	−0.59	−0.10							
Huttunen et al. (2009)	−0.35	0.14	0.12	0.09	0.42	0.91^**^	1.01^***^						
Kumar et al. (2015)	−0.87^***^	−0.38^*^	−0.40^*^	−0.43^**^	−0.10	0.39	0.49	−0.52^**^					
Molla et al. (2019)	−0.88^***^	−0.39	−0.41	−0.44^*^	−0.11	0.38	0.48	−0.53^*^	−0.01				
Shahmahmood et al. (2020)	−0.42^***^	0.07	0.05	0.02	0.35	0.84^**^	0.94^***^	−0.07	0.45^***^	0.46^**^			
Stefanini et al. (2014)	−0.72^***^	−0.23	−0.25	−0.28	0.05	0.54	0.64^*^	−0.37	0.15	0.16	−0.30^*^		
Zhao et al. (2018)	−1.21^***^	−0.72^***^	−0.74^***^	−0.77^***^	−0.44	0.05	0.15	−0.86^***^	−0.34	−0.33	−0.79^***^	−0.49^**^	
Zhumabayev et al. (2022)	−0.69^***^	−0.02	−0.22	−0.25	0.08	0.57	0.67^*^	−0.34	0.18	0.19	−0.27^*^	0.03	0.52^***^

Mean differences are reported as row minus column.

* *p* < 0.05;

** *p* < 0.01;

*** *p* < 0.001.

For the Effects of Implantation subscale, the omnibus ANOVA test was significant, *F*(13,949) = 19.17, *p* < 0.001. Pairwise mean differences between samples are shown in Table 9. Scores in Damen et al. (2007), Anne et al. (2021), Archbold et al. (2008), Brewis et al. (2020), Huttunen et al. (2009), and Shahmahmood et al. (2020) tended to exceed those in Fortunato-Tavares et al. (2012), Stefanini et al. (2014), de Almeida et al. (2015; <24 months and >24months), Kumar et al. (2015), Zhao et al. (2018), and Molla et al. (2019).

**Table 9:** Effects of implantation subscale Tukey HSD *post-hoc* mean differences.

	Anne et al. (2021)	Archbold et al. (2008)	Brewis et al. (2020)	Damen et al. (2007)	de Almeida et al. (2015) < 24 months	de Almeida et al. (2015) > 24 months	Fortunato-Tavares et al. (2012)	Huttunen et al. (2009)	Kumar et al. (2015)	Molla et al. (2019)	Shahmahmood et al. (2020)	Stefanini et al. (2014)	Zhao et al. (2018)
Archbold et al. (2008)	−0.07												
Brewis et al. (2020)	−0.06	0.01											
Damen et al. (2007)	0.14	0.21	0.20										
de Almeida et al. (2015) < 24 months	−0.78	−0.71	−0.72	−0.92^*^									
de Almeida et al. (2015) > 24 months	−0.91^*^	−0.84^*^	−0.85^*^	−1.05^***^	−0.13								
Fortunato-Tavares et al. (2012)	−0.80^*^	−0.73^*^	−0.74^*^	−0.94^***^	−0.02	0.11							
Huttunen et al. (2009)	0.13	0.20	0.19	−0.01	0.91^*^	1.04^**^	0.93^**^						
Kumar et al. (2015)	−0.44	−0.37	−0.38	−0.58^***^	0.34	0.47	0.36	−0.57^*^					
Molla et al. (2019)	−0.74^***^	−0.67^***^	−0.68^**^	−0.88^***^	0.04	0.17	0.06	−0.87^***^	−0.30				
Shahmahmood et al. (2020)	0.26	0.33^***^	0.32^*^	0.12	1.04^**^	1.17^***^	1.06^***^	0.13	0.70^***^	1.00^***^			
Stefanini et al. (2014)	−0.48^*^	−0.41^*^	−0.42^*^	−0.62^***^	0.30	0.43	0.32	−0.61^***^	−0.04	0.26	−0.74^***^		
Zhao et al. (2018)	−0.53^*^	−0.46^**^	−0.47^*^	−0.67^***^	0.25	0.38	0.27	−0.66^***^	−0.09	0.21	−0.79^***^	−0.05	
Zhumabayev et al. (2022)	−0.40	−0.33	−0.34	−0.54^***^	0.38	0.51	0.40	−0.53^**^	0.04	0.34	−0.66^***^	0.08	0.13

Mean differences are reported as row minus column.

* *p* < 0.05;

** *p* < 0.01;

*** *p* < 0.001.

For the Supporting the Child subscale, the omnibus ANOVA test was significant, *F*(14,976) = 43.36, *p* < 0.001. Table 10 displays pairwise mean differences between samples. Byckova et al. (2018), Huttunen et al. (2009), Kumar et al. (2015), and Zhumabayev et al. (2022) tended to support more positive QoL on this subscale compared to the other samples. Also, Brewis et al. (2020) reported significantly higher values than Archbold et al. (2008), Fortunato-Tavares et al. (2012), de Almeida et al. (2015; <24mo and >24mo), Molla et al. (2019), and Shahmahmood et al. (2020).

**Table 10:** Supporting the child subscale Tukey HSD *post-hoc* mean differences.

	Anne et al. (2021)	Archbold et al. (2008)	Brewis et al. (2020)	Byckova et al., 2018	Damen et al. (2007)	de Almeida et al. (2015) < 24 months	de Almeida et al. (2015) > 24 months	Fortunato-Tavares et al. (2012)	Huttunen et al. (2009)	Kumar et al. (2015)	Molla et al. (2019)	Shahmahmood et al. (2020)	Stefanini et al. (2014)	Zhao et al. (2018)
Archbold et al. (2008)	−0.24													
Brewis et al. (2020)	0.19	0.43^**^												
Byckova et al., 2018	0.62^**^	0.86^***^	0.43											
Damen et al. (2007)	−0.14	0.10	−0.33	−0.76^***^										
de Almeida et al. (2015) < 24 months	−0.99^*^	−0.75	−1.18^***^	−1.61^***^	−0.85^*^									
de Almeida et al. (2015) > 24 months	−1.04^**^	−0.80^*^	−1.23^***^	−1.66^***^	−0.90^**^	−0.05								
Fortunato-Tavares et al. (2012)	−1.08^***^	−0.84^**^	−1.27^***^	−1.70^***^	−0.94^***^	−0.09	−0.04							
Huttunen et al. (2009)	0.54^*^	0.78^***^	0.35	−0.08	0.68^***^	1.53^***^	1.58^***^	1.62^***^						
Kumar et al. (2015)	0.43	0.67^***^	0.24	−0.19	0.57^**^	1.42^***^	1.47^***^	1.51^***^	−0.11					
Molla et al. (2019)	−0.78^***^	−0.54^*^	−0.97^***^	−1.40^***^	−0.64^***^	0.21	0.26	0.30	−1.32^***^	−1.21^***^				
Shahmahmood et al. (2020)	−0.79^***^	−0.55^***^	−0.98^***^	−1.41^***^	−0.65^***^	−0.20	−0.25	0.29	−1.33^***^	−1.22^***^	−0.01			
Stefanini et al. (2014)	−0.19	0.05	−0.38	−0.81^***^	−0.05	0.80	0.85^*^	0.89^**^	−0.73^***^	−0.62^**^	0.59^*^	0.60^***^		
Zhao et al. (2018)	−0.14	0.10	−0.33	−0.76^***^	0.00	0.85	0.90^*^	0.94^**^	−0.68^**^	−0.57^**^	0.64^**^	0.65^***^	0.05	
Zhumabayev et al. (2022)	0.60^**^	0.84^***^	0.41	−0.02	0.74^***^	1.59^***^	1.64^***^	1.68^***^	0.06	0.17	1.38^***^	1.39^***^	0.79^***^	0.74^***^

Mean differences are reported as row minus column.

* *p* < 0.05;

** *p* < 0.01;

*** *p* < 0.001.

## Discussion

Overall, parents of children with CIs worldwide assess their child’s well-being positively across multiple domains of CI-specific QoL. Communication and Social relations emerged as the most positively rated aspects of QoL in pediatric CI users globally. In contrast, subscales centered on Effects of implantation and Supporting the child tended to receive the least positive ratings of QoL across studies. Differences across countries in parent proxy ratings of QoL arose across all subscales.

### Comparison with previous literature

Our results converge with previous reports of parent proxy appraisal of QoL in pediatric CI users both for overall well-being and specific domains across a variety of instruments focused on psychosocial well-being. Most of the studies include parental ratings that equaled or exceeded a value of three on a five-point scale, supporting neutral to positive outcomes after cochlear implantation. The two most positively rated domains include Communication outcomes and Social relations, generally followed by Self-reliance and General functioning, in accordance with multiple published papers over the past two decades (Incesulu et al., 2003; Zaidman-Zait et al., 2015). Finally, similar to other studies using the *CCIPP* questionnaire, the subscales centered on Effects of implantation and Supporting a child with CIs tended to receive less positive ratings of QoL, with four samples from two countries—Brazil and Bangladesh—reporting mean scores below three on both subscales (Fortunato-Tavares et al., 2012; de Almeida et al., 2015; Molla et al., 2019).

Visual inspection of parent proxy ratings relative to demographic characteristics (see Table 1) revealed a few subcategory trends in specific QoL domains. Two of the three lowest scores on the social relations domain coincided with studies with the youngest mean age at implantation (Zhao et al., 2018; Shahmahmood et al., 2020). Longer mean duration of CI experience (i.e., ≥4 years) cooccurred with higher parental ratings on the general functioning (Kumar et al., 2015; Brewis et al., 2020; Shahmahmood et al., 2020; Anne et al., 2021) and self-reliance domains (de Almeida et al., 2015; Kumar et al., 2015; Brewis et al., 2020; Anne et al., 2021). This finding echoes previous reports of more positive generic (Huber, 2005; Clark et al., 2012) and CI-specific QoL (Yorgun et al., 2015) with more device experience. No conclusive patterns were observed between mean chronologic age and any *CCIPP* domains. Most studies included in this paper either did not include or did not specify inclusion or exclusion of children with additional comorbidities, although one-third of the sample in Huttunen et al. (2009) had additional disabilities and Anne et al. (2021) included a separate group with developmental disabilities (not included in these analyses), precluding generalization to broader populations of pediatric CI users.

### Value of country comparison

Although many studies have been conducted internationally using the *CCIPP*, this is the first to integrate those findings. Cross-study comparisons can reveal meaningful QoL differences between societies around the world. In our analysis of summary data from 14 studies representing 9 languages and 11 countries, we found significant differences between samples on each of the *CCIPP* subscales.

Significant QoL differences between countries may stem from a variety of factors. Although beyond the scope of the summary data provided in the articles we analyzed, we can speculate on culture as one possible explanation for QoL differences between countries using Hofstede’s Cultural Dimensions Theory. This framework characterizes modern nations according to multiple facets, including individualism versus collectivism (Hofstede, 2003; see Supplementary Table 1 for ratings of individualism and collectivism for each country included in the analyses).

Relative to cochlear implantation, individualistic societies may emphasize specific achievements of the child (e.g., communication) whereas collectivist societies may more highly value fitting in to the group. In general, parents from collectivist societies (e.g., China, Bangladesh) tended to rate their child’s QoL significantly less positively compared to parents from individualistic societies (Zhao et al., 2018; Molla et al., 2019). Studies from highly individualistic communities such as the United States, the United Kingdom, and the Netherlands reported significantly more positive ratings, specifically for domains such as education (Anne et al., 2021) and effects of implantation (Damen et al., 2007; Archbold et al., 2008; Anne et al., 2021) – domains that highlight personal achievement.

Although education traditionally tends to be ranked relatively low among QoL indicators (Hyde et al., 2010; Zhao et al., 2018; Bhamjee et al., 2019), global parent proxy reports using the *CCIPP* ranged considerably from ranking education and academics as the second lowest level of QoL (Kumar et al., 2015; Zhumabayev et al., 2022) to the second highest level of QoL (Molla et al., 2019; Anne et al., 2021) across the subscales. Differences in appraising cochlear implantation relative to academics could indicate country-specific differences in the emphasis on academics in Bangladesh (Molla et al., 2019), Kazakhstan (Zhumabayev et al., 2022), and the United States (Kumar et al., 2015; Anne et al., 2021).

Supporting the child also emerged as a subscale with poor parental ratings, with more than 70% of the included studies ranking it as one of the least positive domains. This finding echoes trends in the literature on pediatric CI users across a wide array of QoL measures (Sach and Whynes, 2005; Stefanini et al., 2014; Zaidman-Zait et al., 2015; Dev et al., 2018; Scarinci et al., 2018). In contrast, parents in three studies using the *CCIPP* appraise supporting the child as one of their top three most positive domains. These three studies tended toward earlier age at implantation and toward the collectivistic side of Hofstede’s scale (i.e., scores between 20 and 60), meaning the well-being of the group takes precedence over the needs of individual persons. Thus, parents in these samples may experience and value support from friends and family regarding their child’s CI.

The individualism–collectivism comparison appears to suggest that pediatric CI users in individualistic societies have more positive QoL, according to parents. However, this conclusion may be dependent on the composition of the *CCIPP* subscales. Archbold and colleagues developed the *CCIPP* based on input from parents in the United Kingdom, a highly individualistic society. As such, the domains that emerged as most important may reflect individualistic priorities rather than underscoring the relevance of more communal aspects of children’s lives, such as family cohesion, a dominant value of close relationships within a member group such as a family in collectivist societies. Collectivist cultural orientations value connection, cooperation, and cohesion within families (Skillman, 2000). Though these facets of QoL are not emphasized in the *CCIPP* or other CI-specific measures, close and responsive family relationships are beneficial for children overall (Boyer and Nelson, 2015) and specifically for children with CIs (Moeller, 2007; Quittner et al., 2013). Thus, it is important to recognize the limitations of our QoL measures, which may not fully capture QoL of children in more collectivist societies.

Aside from Hofstede’s Cultural Dimensions Theory, there are other aspects of society that may explain cross-country differences in QoL. For instance, Finland regularly ranks in the top 10 countries with the highest quality of life with consideration of health, wealth, comfort, safety, and necessities, which may partially explain the high QoL ratings across subscales in the Huttunen et al. (2009) study compared to other papers (Numbeo, 2022; Review, 2022). Additionally, worldwide immigration patterns have led to most countries becoming multicultural. Thus, comparisons of QoL across countries provide a preliminary view of differences that may, in part, reflect cultural values. The current study quantifies mean differences in QoL of children with CI that have been reported across countries around the world as a first step that will hopefully inspire future research on variations in cultural values both between and within countries.

### Clinical implications of country comparisons

Societal differences can be reflected in dynamics of parent–child interaction, with cascading effects on a parent’s accuracy in appraising their child’s QoL. A parent’s attunement to their child’s well-being may be affected by the parent–child attachment relationship (Bowlby, 1978; Weiss, 1991) and cultural expectation, which can guide aspects of parenting including discipline and social behavior.

Attachment theory suggests more attuned parents can report their child’s wellness more accurately than less attuned parents (Ainsworth, 1967; Ma and Huebner, 2008). Secure attachment may explain parent attunement within individualistic cultures, but may be a less precise predictor in collectivistic cultures where children could develop emotional bonds through multiple caregiving arrangements, sharing secure attachments with adults outside of their parents (e.g., grandparents, aunts, older siblings; Keller, 2012, 2017). This means that accurate assessment of well-being in children with CIs may require collection of proxy ratings from multiple sources rather than a single parental rating.

Cultural norms also shape parental expectations in areas such as perceptual progress, cognitive achievements, and developmental timetables. Studies from Israel, Japan, the United States, and Australia found differences in expectations in milestones for vision, audition, and language development (i.e., receptive and expressive; see Williams et al., 2000, for a review of the literature). These differences indicate potential biases in our understanding of child development, which predominantly stems from research guided by Western cultural beliefs (Escovar and Lazarus, 1982; Sahithya et al., 2019), and may also influence response patterns on parent-report questionnaires.

Relative to QoL, identifying areas where cultural expectations may differ could better explain why parents rated domains more or less positively (e.g., communication outcomes). Families from individualistic cultures may expect reciprocal conversation, with equal turn-taking between parent and child, whereas families from collectivistic cultures may use more parent-driven interactions in which children take a more passive role in conversations (Ochs, 1988; Bornstein, 2007, 2012, 2013). Items on the *CCIPP* subscales may emphasize individualistic values (e.g., Communication: *Communication is difficult even with people he knows well* and *Now he is talkative and engages others in conversation*; Social relations domain: *He does not have a close relationship with his grandparents* and *He shares in family situations more than before implantation*.). Thus, current instruments measuring condition-specific QoL may not accurately reflect the experiences of children with CIs from other cultures, necessitating more comprehensive understanding of similarities and differences in expected outcomes across societies (Sahithya et al., 2019).

### Strengths and limitations

This study has strengths in that it marks the first study to explore differences across samples collected in countries around the world using the same CI-specific QoL instrument, the *CCIPP*. Restricting study inclusion to articles using the same measure allows for direct comparison of results across the various subscales in a multitude of countries. In addition, understanding expected outcomes by country could help to provide customized services or counseling to children and their families from another country or culture.

Despite these strengths, this study has an important limitation. We collected summary data from previously published papers that varied in the level of detail provided on sample characteristics. Conducting a retrospective study allowed for a first attempt to compare parent-reported CI-specific QoL, but it introduced a lack of control over the subject population (e.g., chronologic age, implantation age, device experience, auditory performance, therapeutic intervention, absence of additional disabilities). Future studies should enlist a prospective approach with a controlled subject population to allow more comprehensive understanding of the independent and interdependent influence of cultural background and values, socio-economic status, parenting beliefs, child performance, etc. on QoL ratings among children with CI.

## Conclusion

Understanding QoL differences across societies around the world can offer insight into where a child with CI struggles or succeeds, enabling health care professionals, researchers, and clinicians to deliver the most meaningful services to children with CIs and their parents. This may require practitioners to delve into the literature to better understand the dynamic relations among culture, parental expectations, and child development within the sociocultural context (Bornstein, 2012).

Specific to pediatric cochlear implantation, understanding similarities and differences in QoL across societies could be useful when providing services to children from another country or culture who are acclimating to a new environment. Awareness of QoL domains that parents rate less positively can encourage appropriate programming and services to bolster well-being in particular domains. Parents can be better equipped to anticipate potential psychosocial outcomes their children could face socially, academically, and psychologically at school and home. Proactive preparation could ease CI-specific transitional difficulties and hopefully decrease distressing situations for children with CI. In efforts to generate greater life satisfaction for children with CI, the worldwide gap between children with typical hearing and children with CIs could become less noticeable over time.

In conclusion, global parent proxy ratings of QoL in pediatric CI users overall reflect positive outcomes across multiple domains, particularly in the areas of communication and social interactions. However, absolute and relative appraisal of well-being significantly differed across the studies from different countries and cultures. These findings highlight the fact that QoL reflects a multi-factorial construct based not only on demographic characteristics of the child, but also of the family and the broader sociocultural environment. Clinicians should strive to respectfully incorporate culturally sensitive practices into therapeutic intervention to maximize well-being in ways that matter to individual families and their children with CIs.

## Data availability statement

The original contributions presented in the study are included in the article/Supplementary material, further inquiries can be directed to the corresponding author.

## Author contributions

AW-C, JN, and RK contributed to the conception and design of the study. AW-C accumulated the articles and established the database. JN performed the statistical analyses. AW-C and RK wrote the initial draft of the manuscript as part of RK’s thesis. AW-C wrote the first draft of the manuscript with JN, RK, and SC each contributing unique sections. All authors contributed to the article and approved the submitted version.

## Funding

The authors thank Beth and Mohammed Anis, whose private donation to support the research of the AW-C covered the open access publication fees.

## Conflict of interest

The authors declare that the research was conducted in the absence of any commercial or financial relationships that could be construed as a potential conflict of interest.

## Publisher’s note

All claims expressed in this article are solely those of the authors and do not necessarily represent those of their affiliated organizations, or those of the publisher, the editors and the reviewers. Any product that may be evaluated in this article, or claim that may be made by its manufacturer, is not guaranteed or endorsed by the publisher.

## Footnotes

^1^ https://statpages.info/anova1sm.html
