# Supplementary material for: Parent-reported quality of life in children with cochlear implants differs across countries
Source: Front Psychol. 2022 Oct 6;13:966401. doi: 10.3389/fpsyg.2022.966401 (PMC9583949; doi:10.3389/fpsyg.2022.966401)

Supplementary Figures 1–8

Means plots for each subscale

Warner-Czyz, A.D., Nelson, J.A., Kumar, R., & Crow, S. (2022). Parent-reported quality of life in children with cochlear implants differs across countries. *Frontiers in Psychology*. doi: 10.3389/fpsyg.2022.966401

Figure 1.

Means Plot for Communication

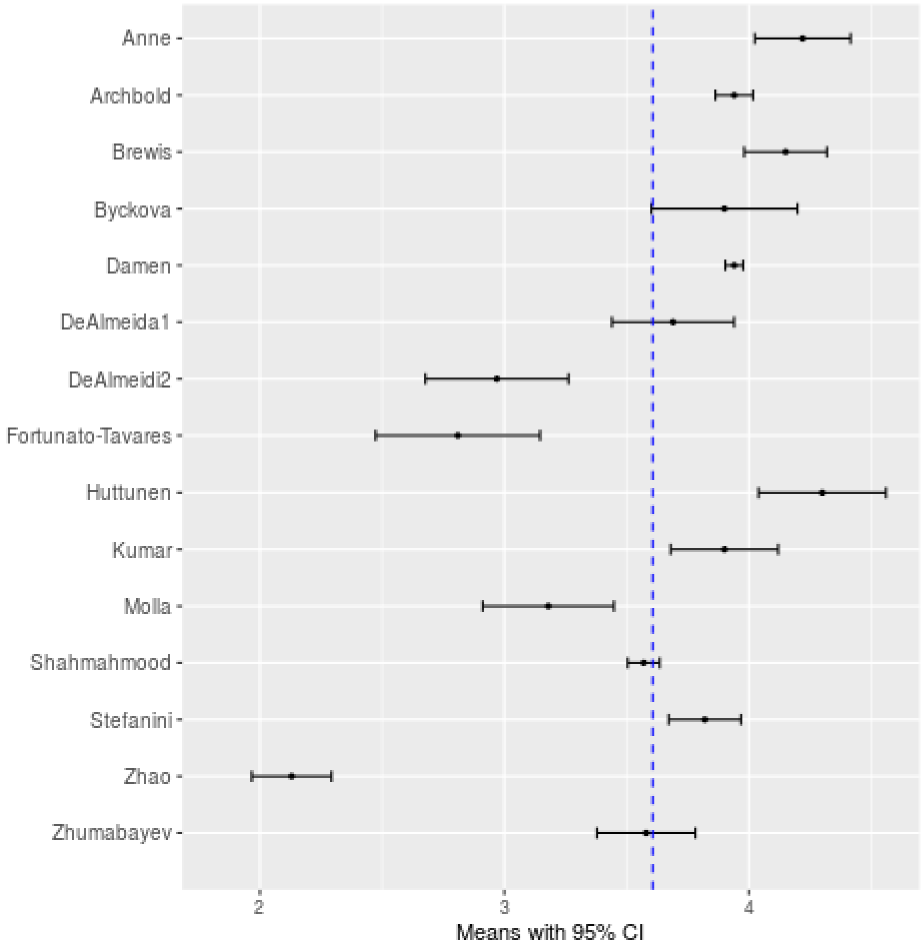

Figure 2.

Means Plot for General Functioning

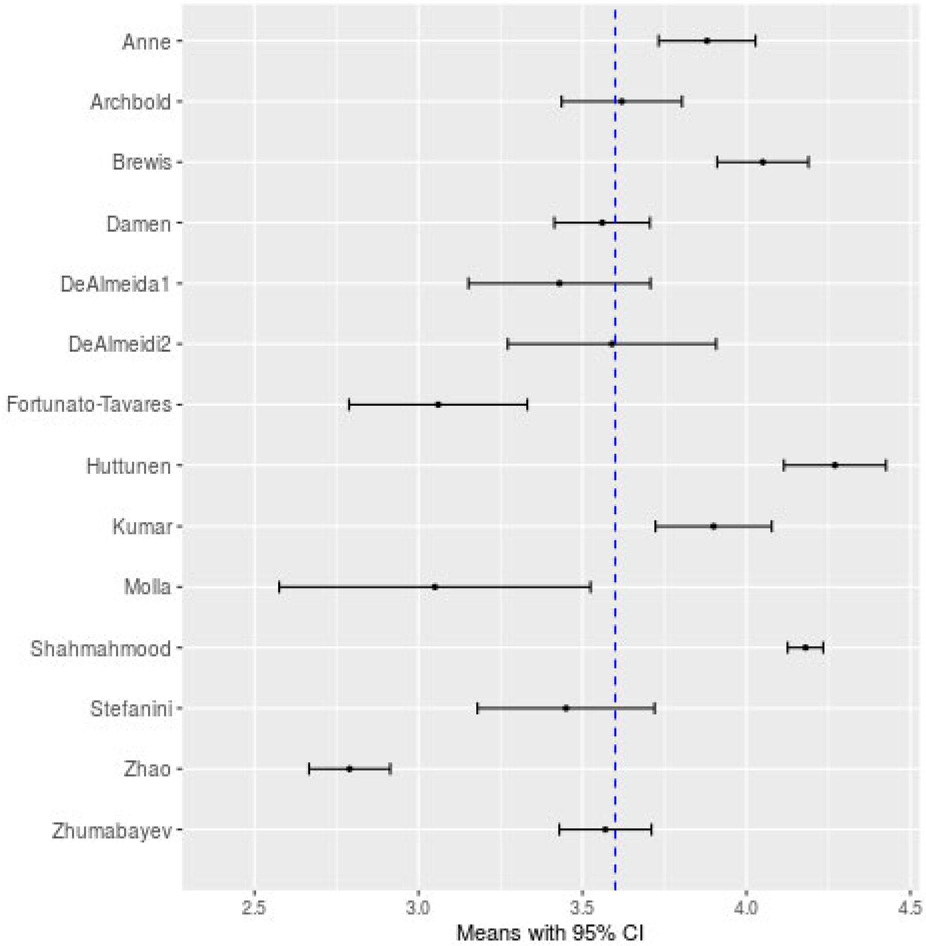

Figure 3.

Means Plot for Self-Reliance

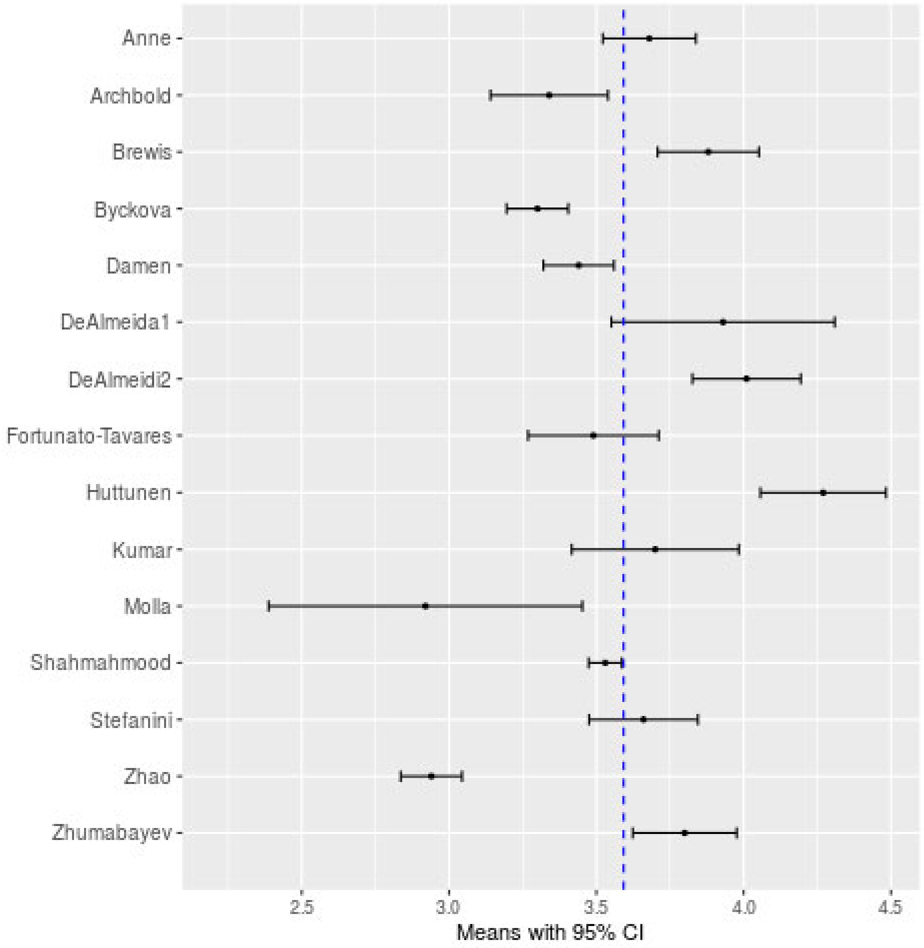

Figure 4.

Means Plot for Well-Being

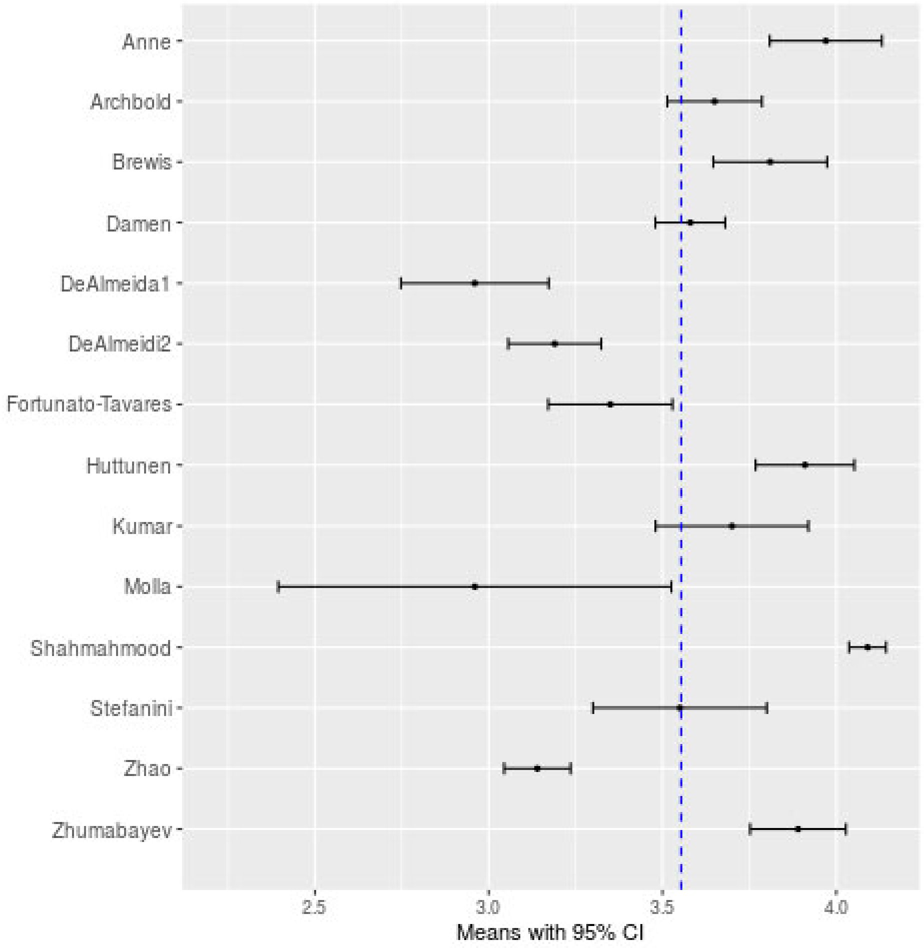

Figure 5.

Means Plot for Social Relations

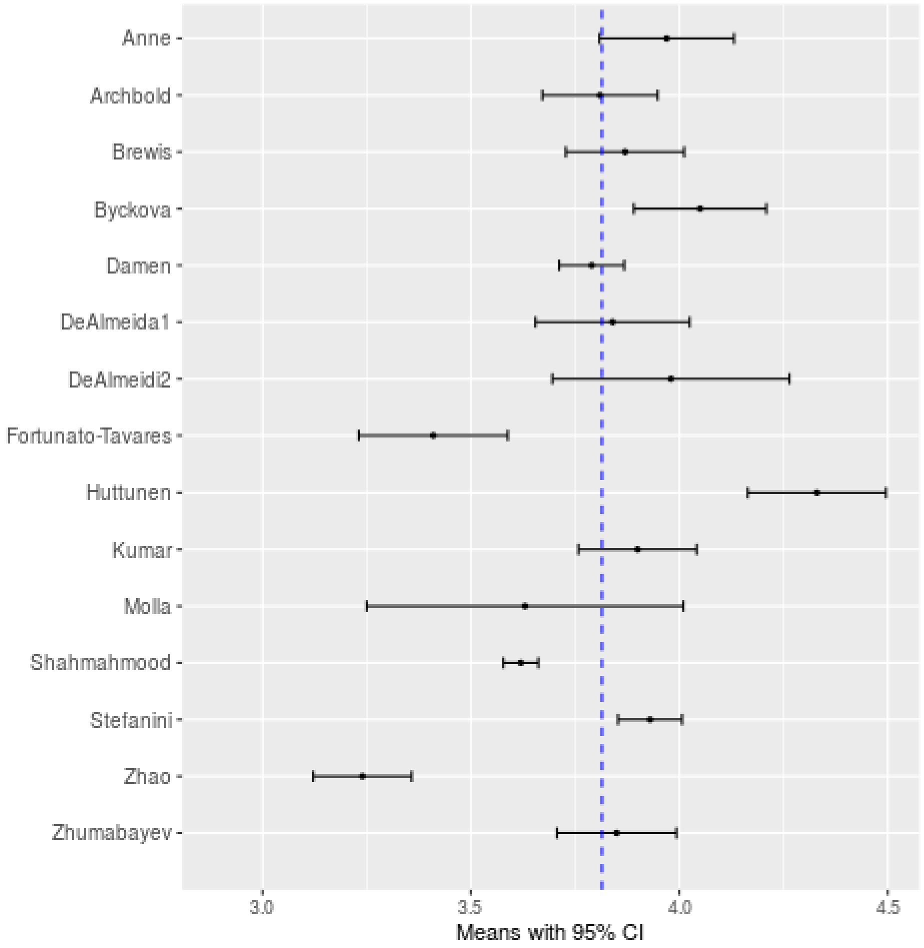

Figure 6.

Means Plot for Education

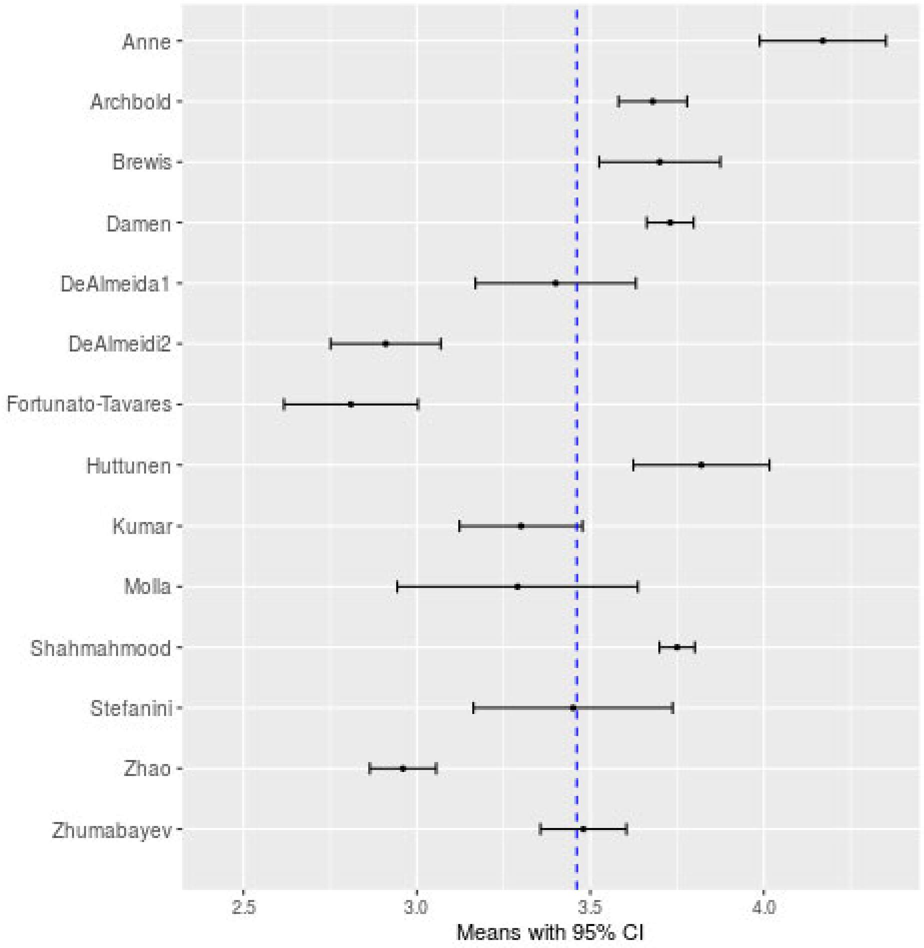

Figure 7.

Means Plot for Effects of Implantation

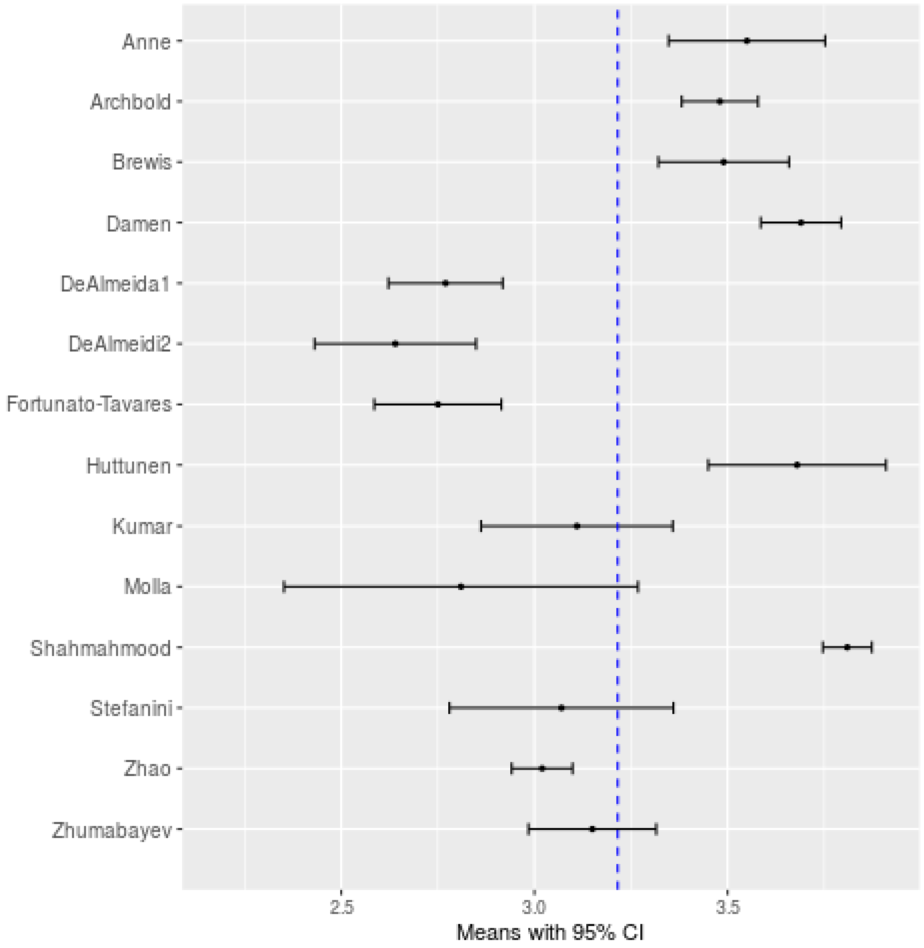

Figure 8.

Means Plot for Supporting the Child

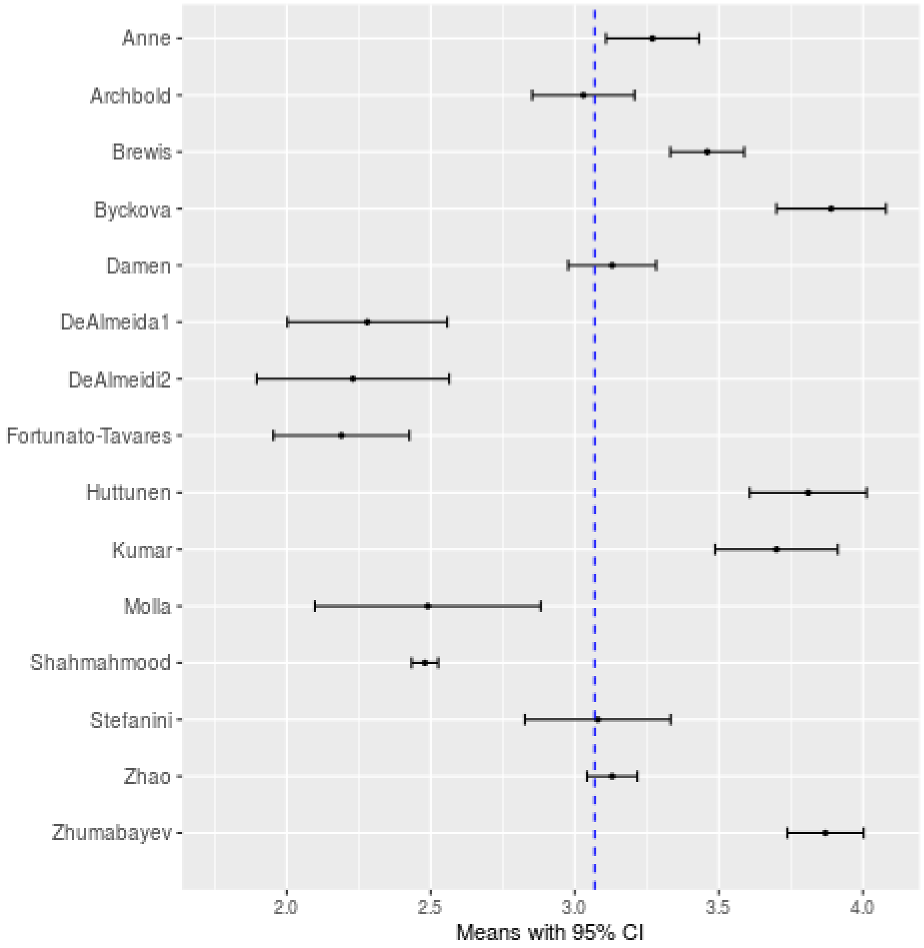

Supplement: Supplementary file 1 [file Presentation_1.pdf]
