# Supplementary material for: Parent-reported quality of life in children with cochlear implants differs across countries
Source: Front Psychol. 2022 Oct 6;13:966401. doi: 10.3389/fpsyg.2022.966401 (PMC9583949; doi:10.3389/fpsyg.2022.966401)
Supplement: Supplementary file 2 [file Table_1.docx]

Supplementary Table. Summary of articles, countries, and Hofstede individualism-collectivism score.

| Author | Country | Hofstede individualism-collectivism rating |
| --- | --- | --- |
| Molla (2019) | Bangladesh | 20 |
| Zhao (2018) | China | 20 |
| Zhumbayev et al. (2022) | Kazakhstan | 20 |
| de Almeida et al. (2014)  Fortunato-Tavares et al. (2012)  Stefanini (2014) | Brazil | 38 |
| Shahmahmood et al. (2020) | Iran | 41 |
| Byčkova et al. (2018) | Lithuania | 60 |
| Huttunen et al. (2009) | Finland | 63 |
| Brewis et al. (2020) | South Africa | 65 |
| Damen et al. (2007) | Netherlands | 80 |
| Archbold et al. (2008) | UK | 89 |
| Anne et al. (2021)  Kumar et al. (2015) | USA | 91 |

*Note.* The Hofstede individualism-collectivism rating derives from Hofstede’s Cultural Dimensions Theory, a framework for cross-cultural comparison (Hofstede, 2003). The individualism versus collectivism spectrum reflects the extent to which importance centers on the goals and well-being of a specific person (i.e., individualism) or the goals and well-being of the group (i.e., collectivism). The scale spans from 0 to 100, with lower scores coinciding with collectivistic values and higher scores corresponding to individualistic values.
